# Chemical Signaling and Integrated Strategies for Biological Control in Crop Agroecosystems

**DOI:** 10.3390/insects17080808

**Published:** 2026-08-04

**Authors:** Shakil Ahmad, Dasong Chen, Yongjie Cen, Momana Jamil, Hina Gul, Valeria Palma-Onetto, Gecheng Ouyang, Meng Xiang

**Affiliations:** 1 Guangdong Key Laboratory of Animal Conservation and Resource Utilization, Institute of Zoology, Guangdong Academy of Science, Guangzhou 510260, China; mshakil.aup@gmail.com (S.A.); chendasong@gmail.com (D.C.); 20220295@m.scnu.edu.cn (Y.C.); ouyanggc@giz.gd.cn (G.O.); 2 School of Ecology, Hainan University, Haikou 570228, China; jamilmomana@gmail.com; 3 State Key Laboratory for Quality and Safety of Agro-Products, Institute of Plant Protection and Microbiology, Zhejiang Academy of Agricultural Sciences, Hangzhou 310021, China; gulhina680@gmail.com; 4 Departamento de Química y Medio Ambiente, Universidad Técnica Federico Santa María, Avenida España 1680, Valparaíso 2390123, Chile; valeria.palmao@usm.cl

**Keywords:** herbivore-induced plant volatiles (HIPVs), natural enemies, chemical ecology, plant–insect interactions, indirect defense, integrated pest management (IPM)

## Abstract

Food security faces significant challenges due to the growing threat of insect pests and global climate change, making it essential to adopt sustainable agricultural strategies. Plants possess intrinsic defense systems that are activated in response to herbivore attack, including the emission of specialized volatile organic compounds (VOC) known as herbivore-induced plant volatiles (HIPVs). These volatiles aid the foraging of natural enemies (predators and parasitoids), thereby contributing to biological control of pest populations. Harnessing these chemical defense mechanisms offers a promising avenue for pest management, although natural enemy attraction does not necessarily result in pest suppression or increased crop yield. This review synthesizes plant and insect-derived chemical signaling mechanisms with direct relevance to biological control in crop agroecosystems, including field crops, orchards, vegetable systems, and protected cultivation. We emphasize recent applied research while retaining selected foundational studies that established the principal concepts and mechanisms underlying chemically mediated pest management. Considering the importance of chemical signaling in sustainable agriculture, we propose four key research directions: (1) optimizing plant defense induction mechanisms; (2) integrating HIPVs with natural enemy pheromone-based attractants; (3) combining habitat management with the provision of plant-derived food resources; and (4) harnessing plant defenses through breeding and infochemical-based genetically modified crops. Additionally, we examine the potential impacts of environmental stressors on plant–herbivore chemical communication, underscoring their importance for resilient and environmentally sustainable agricultural systems.

## Simple Summary

Insect pests cause serious losses in many crops, and their control often depends heavily on chemical pesticides. Although pesticides can be effective, their repeated use may create environmental risks and can also affect beneficial insects that naturally help control pests. Plants, however, have their own ways of defending themselves. When attacked by herbivorous insects, many plants release specific odors that can be detected by predators and parasitoids. These beneficial insects use herbivore-induced plant volatiles, including terpenoids, green leaf volatiles, and compounds such as methyl salicylate, to locate pest-infested plants and reduce pest populations. This review explains how plant odors, insect pheromones, and other chemical cues can be used to improve biological control in agriculture. It also discusses how these signals can be combined with habitat management, flowering plants, push–pull systems, attract-and-reward strategies, plant breeding, and genetically engineered crops. Together, these approaches may support natural enemy conservation, although their effects on pest suppression, crop yield, and pesticide use must be confirmed under field conditions.

## 1. Introduction

The food crisis has become a severe issue as the global population grows and resources for food production decline [1,2]. One of the primary issues is the increasing outbreaks of insect pests and associated crop losses [3], threatening food production and placing considerable pressure on efforts to feed a growing world population [4]. Increasing damage caused directly by herbivory and indirectly through pathogen transmission poses significant risk to global food security [5,6] and biodiversity loss [7]. According to estimates by the Food and Agriculture Organization of the United Nations (FAO), the global population is expected to reach 9.7 billion by 2050, requiring a 70% increase in food production to address the anticipated food demands [8,9]. Therefore, improved knowledge of sustainable food production strategies is needed to meet the growing need for food. Sustainable pest management strategies are therefore essential to reduce crop losses while minimizing environmental impacts.

**Figure 1:**
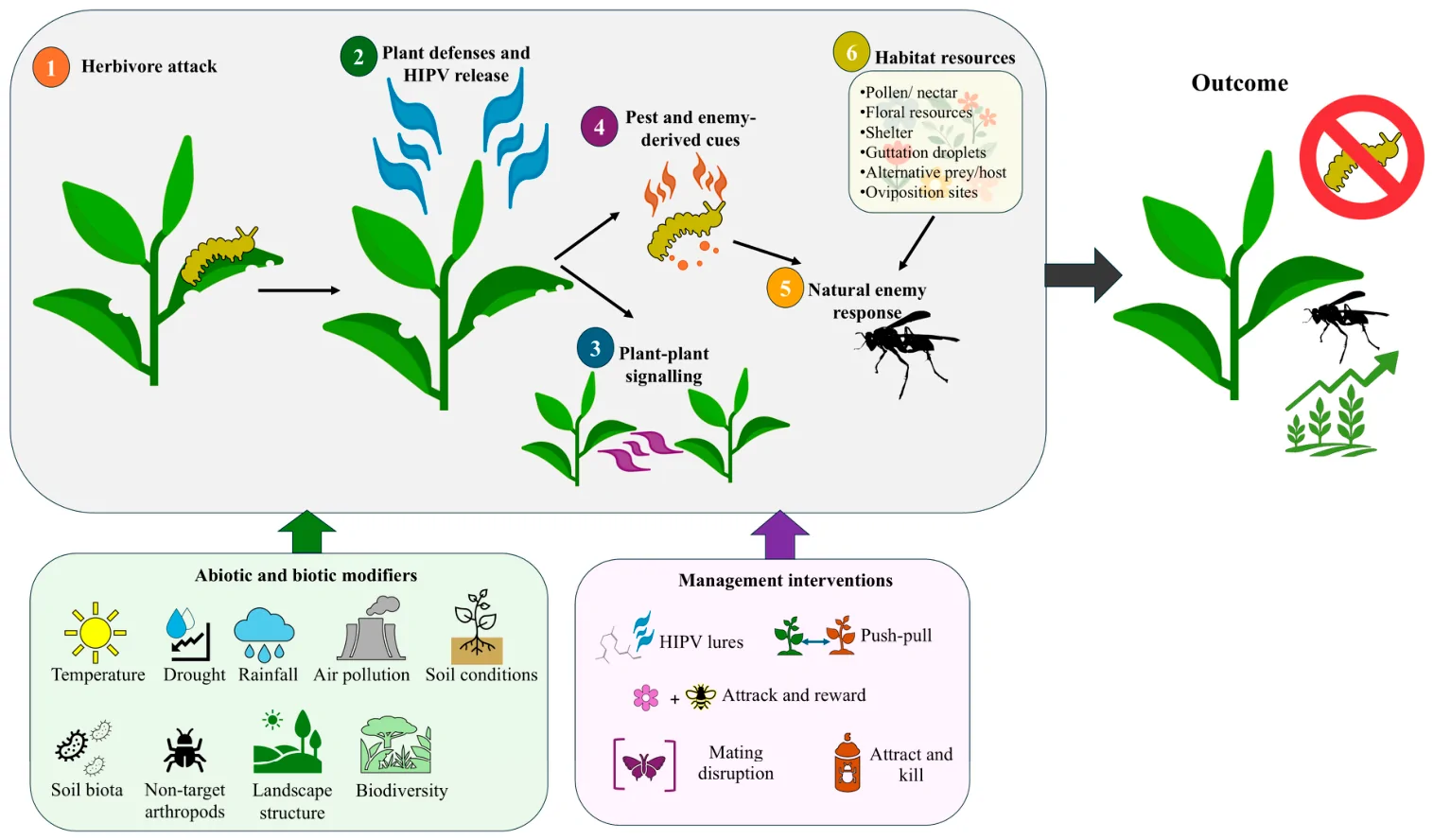
Conceptual framework of chemically mediated biological control in crop agroecosystems. Herbivore attack induces plant volatiles and other chemical cues that influence plant signaling, natural enemy recruitment, and pest behavior. Habitat resources, environmental factors, and semiochemical-based management strategies jointly determine pest suppression and crop protection.

Plants rely on unique mechanisms of recognition, signaling and defense to respond to herbivore attack (Figure 1, Component 1) and pathogens that have a negative impact on plant fitness. Defensive strategies include direct and indirect chemical defenses [10,11]. Direct defenses include the production or accumulation of toxic secondary metabolites, defensive proteins, and other compounds that reduce herbivore performance [12]. Indirect defenses involve the emission of herbivore-induced plant volatiles (HIPVs) from herbivore-attacked plants (Figure 1, Component 2), which can recruit natural enemies such as predators and parasitoids [13,14]. The first comprehensive discussion of tritrophic interactions emerged more than four decades ago. In 1980, Peter W. Price introduced the concept of plant traits that had evolved to recruit natural enemies of herbivores by emphasizing on the adaptive significance of these interactions [15]. Many other researchers, such as Bradleigh Vinson, Donald Nordlund, and Joe Lewis, reported the first pieces of evidence of the attractiveness of herbivore-damaged plants to herbivores’ natural enemies [16,17]. For decades, research focused primarily on how predatory mites locate their prey using plant-emitted volatiles [18]. However, in recent years a considerable amount of research has investigated the regulatory mechanisms of plant volatile emissions in plant–plant and plant–arthropod interactions [19,20] and their influence on plant performance, offering promising avenues for sustainable crop protection [21,22].

This review is organized around four interconnected research directions summarized in Figure 1: optimizing plant defense induction mechanisms, including plant–plant signaling (Figure 1, Component 3); integrating HIPVs with pest- and natural-enemy-derived chemical cues (Figure 1, Component 4); combining habitat management with plant-derived food and shelter resources; and harnessing plant defenses through selective breeding and infochemical-based genetic engineering. Given the breadth of chemical ecology, this review is not intended to provide an exhaustive account of all semiochemical-mediated interactions. Instead, it focuses on plant- and insect-derived chemical signals that have direct or emerging relevance to biological control in crop agroecosystems, including field crops, orchards, vegetable production, and protected cultivation. Recent applied studies are emphasized, while selected foundational studies are retained where they are necessary to explain major concepts, mechanisms, and the historical development of the field. Unlike previous reviews focused mainly on individual semiochemicals or specific biological control approaches, this review integrates chemical signaling, habitat management, environmental stressors, and crop improvement strategies within a unified framework for crop agroecosystems.

## 2. Challenges to Global Food Security

Food security remains a major global challenge, with hundreds of millions of people affected by chronic hunger and malnutrition [23]. Crop production is constrained by insect pest outbreaks, climate change, and continued dependence on synthetic pesticides [24,25,26]. Although pesticides remain important for crop protection, intensive use may affect non-target organisms and environmental health [27], supporting the development of complementary and more selective pest management approaches.

Globally, plant pests and diseases are estimated to cause 20–40% losses in crop production each year, including major losses in staple crops such as wheat, rice, maize, soybean, and potato [28]. These pest and disease outbreaks not only cause significant decreases in agricultural yield and product quality but also directly affect biodiversity, food security, and human health [3,29]. Furthermore, climate change poses an increased risk of plant disease intensification by influencing pathogen evolution and host–pathogen interaction which enables the emergence of new pathogenic strains [30]. Moreover, a shift in geographical range of pathogens increases the risk of plant disease spread in unaffected areas, which in turn has a significant negative effect on global food supply and demand as a result of inconsistent supply channels to market, increasing market prices and decreasing food accessibility to vulnerable population [30,31]. Addressing these challenges requires integrated approaches that move beyond conventional pest control methods while maintaining sufficient crop yields. Chemical ecology offers promising tools to reduce reliance on synthetic pesticides by exploiting plant–insect signaling, natural enemy recruitment, and behaviorally based pest management strategies [32,33]. Examples include methyl salicylate lures that attract predators and parasitoids, pheromone-based mating disruption, attract-and-kill systems, and push–pull strategies that repel pests from the main crop while attracting them to trap crops or baited traps.

## 3. Chemical Signals and Their Application in Sustainable Pest Management

### 3.1. Chemical Ecology in Pest Management

Chemical communication is pivotal to modulating key insect behaviors, including reproduction, foraging, territorial marking, recruitment of colony members, and alarm signaling, all of which are essential for their survival. These behaviors are largely mediated by chemical cues known as semiochemicals, which facilitate communication within and between species across trophic levels [34]. These chemical cues hold significant potential in integrated pest management (IPM) to maintain pest population below an economic threshold. Many semiochemicals are relatively species-specific and can influence target insect behavior with reduced toxicity to beneficial organisms compared with conventional insecticides; however, non-target effects on natural enemies may occur depending on the compound and application context. Compared with conventional broad spectrum insecticides, semiochemical-based approaches are generally considered more environmentally benign because they act primarily by modifying insect behavior rather than by direct toxicity. However, their ecological effects can vary depending on compound specificity, dose, formulation, deployment strategy, and potential effects on non-target organisms [34,35]. In the last few decades, numerous subsequent studies have provided a considerable body of information on chemical communication, semiochemicals and their successful application in IPM programs, such as mating disruption, mass trapping, attract-and-kill, and push–pull techniques [36]. For instance, in cabbage production systems in Costa Rica and Nicaragua, pheromone-baited mass trapping of the diamondback moth, *Plutella xylostella*, significantly reduced insecticide applications, increased yields, and improved net profits by enhancing farmer adoption of IPM strategies [37,38]. Additionally, a recent study identified a male-specific aggregation pheromone, 2-isobutyl-3-methoxypyrazine, associated with pistachio leaves, which could support the development of pheromone-based traps or behavioral disruption strategies for the leaf beetle *Labidostomis lusitanica* [39]. While HIPVs are mainly involved in plant-mediated attraction of natural enemies and other trophic interactions, mating disruption relies primarily on insect sex pheromones that interfere with mate location. Together, these approaches illustrate how different classes of semiochemicals can be integrated into IPM [40,41]. Mating disruption is a key pheromone-based IPM strategy, and recent advances have focused on sprayable microencapsulation-based formulations. For example, this approach reduced trap catches of the spruce budworm *Choristoneura fumiferana* by approximately 90%; however, it did not significantly reduce population density, highlighting the need to distinguish behavioral disruption from actual pest suppression [42].

### 3.2. Current Chemical Ecology Approaches for Enhancing Biological Control

Chemical ecology approaches are increasingly being incorporated into biological control to manipulate the behavior of predators and parasitoids in crop systems [43]. HIPVs may provide long-range cues that help natural enemies locate herbivore-infested plants, although their effectiveness varies with the compound, release rate, crop system, and ecological context [44,45]. Two widely studied approaches are the induction of plant volatile emissions using methyl jasmonate and the direct application of attractive compounds such as methyl salicylate [20].

## 4. Integrating Chemical Signals to Enhance Biological Control

### 4.1. Integrating HIPV-Based Strategies to Support Natural Enemies

The attraction and retention of effective natural enemies mediated by semiochemicals are crucial for the success of conservation biological control (CBC) programs in diverse agricultural systems (Figure 1, Component 5). Natural enemies require multiple resources to persist in crop habitats, including prey or hosts, suitable shelter, alternative food sources, oviposition sites, and, in some cases, conspecific cues for reproduction (Figure 1, Component 6). Providing these resources can improve their attraction, retention, and performance in biological control systems. Natural enemy conservation can also be supported through plant breeding by selecting traits that favor natural enemy retention and contribute to herbivore suppression, such as extrafloral nectaries, domatia, and appropriate trichome density [46,47]. However, the significance of information-based indirect defenses has largely dominated in applied research due to their potential in signaling and attracting natural enemies (Figure 1). The following subsections summarize future considerations for leveraging HIPV-based strategies to enhance natural enemy recruitment, retention, and pest suppression in diverse agricultural contexts.

### 4.2. Integrating HIPVs with Insect-Derived Cues for Biological Control

Biological control, delivered by natural enemies, is one of the most promising alternatives to synthetic pesticides for managing herbivorous pests in sustainable crop production. The effectiveness of biological control strategies can be significantly advanced by combining the HIPVs with host insect chemical cues. Recent studies have shown that HIPVs can guide host-searching parasitoids over relatively long distances, whereas low-volatility cuticular hydrocarbons (CHCs) left by target herbivores on plant surfaces can provide short-range contact cues for host location [48]. For instance, integrating HIPVs to attract parasitoids into cropping systems can be further optimized by ensuring the presence of CHCs on the feeding surface left by target pests [49,50,51]. A recent study has identified a specific cuticular compound 1-hexadecene in the stink bug *Halyomorpha halys*. This CHCs is exploited as a contact chemical cue by the egg parasitoid *Trissolcus japonicus*. The female *T. japonicus* exhibited intensive foraging behavior for host eggs and spent more time on surfaces with host-associated chemical traces [52]. By mimicking or amplifying these chemical cues, biological control strategies can improve predator and parasitoid attraction through HIPVs while enhancing short-range arrestment and host-targeting accuracy through CHCs and other herbivore-derived contact cues. Additionally, integrating both conspecific and interspecific chemical cues to encourage natural enemy recruitment is critical for an effective IPM program. For instance, combining HIPVs with kairomones (interspecific cues) has been shown to recruit natural enemies more effectively. In wheat, combining the aphid alarm pheromone (E)-β-farnesene with herbivore-induced MeSA increased parasitism and reduced the abundance of the aphid *Sitobion miscanthi* [53]. Alarm pheromones may also reduce pest colonization by inducing dispersal or avoidance behavior in conspecifics, although their effectiveness depends on pest species, crop context, release rate, and field conditions [54] (Figure 1). In addition to harnessing chemical cues for herbivore colonization reduction, aggregation pheromones hold potential for predator population retention in agriculture systems. For example, combining MeSA with aggregation pheromone lures of the predatory stink bug *Podisus maculiventris* in tomato crop significantly enhanced the augmented predators retention and reduced caterpillar *Manduca sexta* infestation [55]. Because *Podisus maculiventris* is a predatory stink bug rather than a plant-feeding pest, its aggregation pheromone is intended to retain beneficial predators rather than attract herbivorous stink bugs; nevertheless, species specificity and non-target attraction should be evaluated before field deployment. This insect-derived dual-signal approach may improve biological control by combining long-range attraction with short-range host/prey location and retention cues, making pest suppression more reliable under suitable crop and ecological conditions.

### 4.3. Balancing Targeted Recruitment with Natural Enemy Community Resilience

Plants release blends of HIPVs in response to herbivore attack. The composition and quantity of these compounds can vary significantly depending on several factors, such as the herbivore and plant species, developmental stage, environmental conditions and the number of herbivore species infesting the plant [56]. This variability can generate chemically distinct signals that differ in their attractiveness to natural enemies. While increasing natural enemy diversity generally strengthens biological control [57], using blends of HIPVs as a strategy for synergistically attracting a broad range of natural enemies presents challenges [58]. Because natural enemies may respond to specific ratios or combinations of HIPVs, adding multiple compounds without considering blend composition can mask key signals, disrupt signal specificity, or reduce attraction [59]. Moreover, in some cases certain combinations of HIPVs are not only ineffective but can also repel natural enemies and compromise the intended effect of their application.

A more effective approach may be to target functionally important natural enemies while maintaining community-level diversity and functional redundancy [60,61]. Integrating HIPVs with natural enemy pheromones or kairomones could improve recruitment of selected predators or parasitoids [43], but such strategies should complement, rather than replace, a diverse natural enemy community (Table S1). A recent study was conducted in sweet pepper to manage flower thrips, including *Frankliniella occidentalis* and *Frankliniella tritici*, which are important pests of many crops. The study used MeSA, a common HIPV, in combination with the thrips’ aggregation pheromone neryl (*S*)-2-methylbutanoate (NMB), to evaluate attraction of *Frankliniella occidentalis* and its predator, *Orius insidiosus*. The results showed that traps baited with MeSA captured significantly more thrips than traps containing NMB or no lure controls, indicating that HIPV-based lures may influence pest behavior as well as natural enemy responses [62]. However, because pest aggregation pheromones may also increase pest attraction, their use should be carefully evaluated to ensure that enhanced natural enemy recruitment outweighs any potential increase in pest colonization or crop damage. Additionally, kairomone-based lures have been explored to enhance biological control strategies by attracting and retaining natural enemies, thereby reducing insect pest populations [63]. Integrating HIPVs with pheromones or kairomones may improve biological control by combining long-range attraction with more specific cues for host/prey location or predator retention. However, these combined strategies should be designed carefully because some lures may also attract pest species, alter natural enemy behavior unpredictably, or fail to translate attraction into effective pest suppression. Field evaluations should therefore assess not only the response of the targeted species but also effects on natural enemy diversity, interspecific interactions, and the resilience of pest suppression under environmental variation.

### 4.4. Improving the Attract-and-Reward Strategy

**Figure 2:**
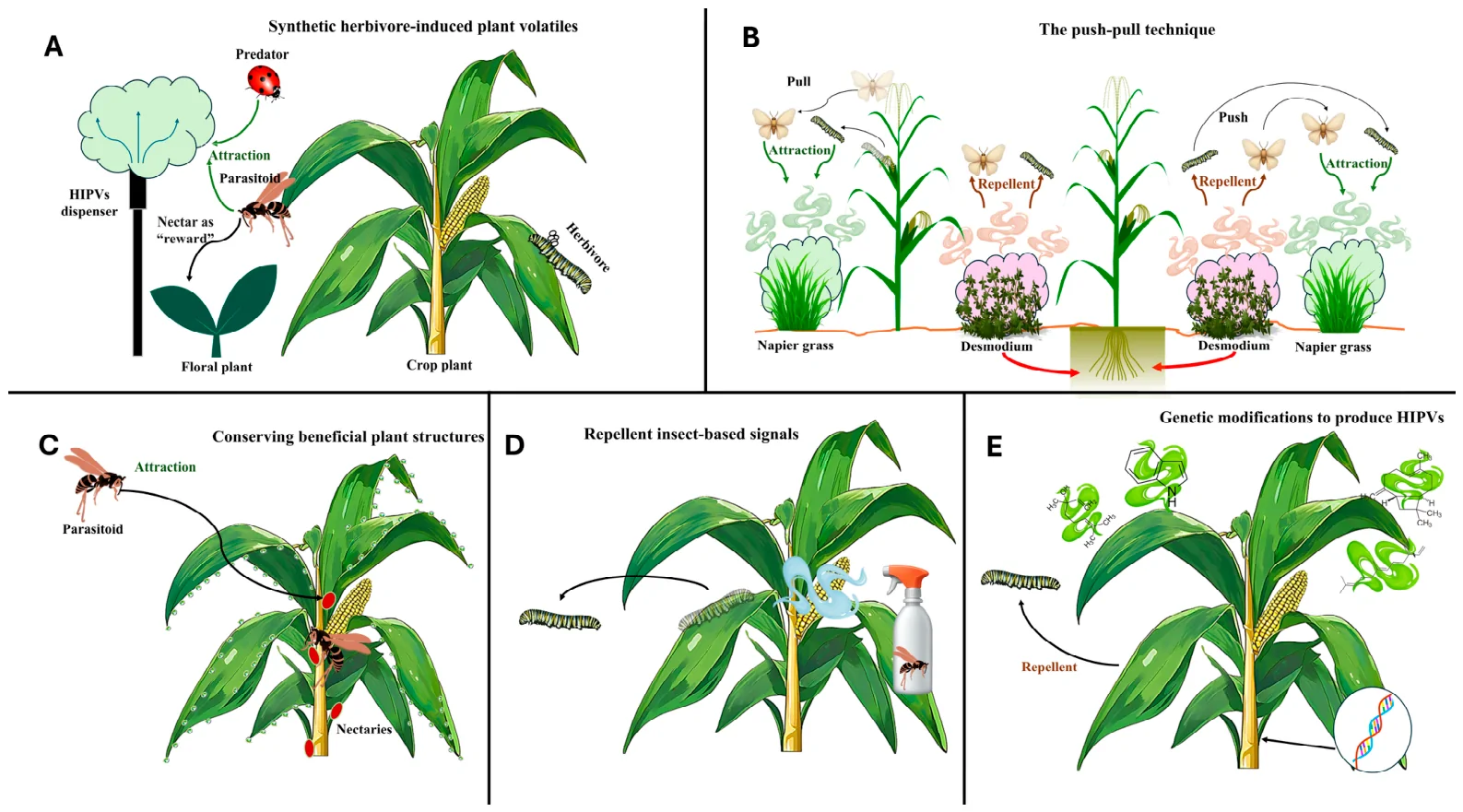
Schematic representation of conservation biological control (CBC) strategies for multifaceted pest management. The attract-and-reward strategy pairs synthetic HIPV dispensers as attractants with floral or other resource providing plants as rewards to support the attraction and retention of natural enemies (**A**). The push–pull technique combines repellent plants or semiochemicals that push insect pests away from the main crop with attractive trap crops or baited traps that pull pests into areas where they can be concentrated and managed (**B**). Conserving plant-derived nutrient-rich food sources, such as guttation, and beneficial plant structures can help retain natural enemies within cropping systems (**C**). Pest pheromone-based mating disruption approaches interfere with pest mate location, reducing reproduction and colonization (**D**). Infochemical-based genetically modified crops may enhance natural enemy attraction while repelling or suppressing herbivores (**E**).

The attract-and-reward strategy has been used for decades to increase natural enemy abundance in crops, with the aim of improving pest population suppression. This approach emphasizes natural enemy retention in cropping system and may serve as a vital component of IPM across many agricultural systems. By using synthetic HIPVs, natural enemies can be attracted from surrounding habitats into the main crop and retained with rewards such as floral plantings, nectar, pollen, shelter, or alternative prey/hosts when target pest populations are low or temporarily absent (Figure 2A) [64,65]. Studies have demonstrated the effectiveness of attract-and-reward combinations in brassica, sweet corn, wine grape, and broccoli systems using buckwheat, *Fagopyrum esculentum*, as the reward plant and MeSA as the volatile attractant [66]. This strategy significantly increased the abundance of parasitoids, including tachinid flies and species such as *Microplitis demolitor*, *Trichogramma pretiosum*, and *Dolichogenidea tasmanica*, and helped retain their populations over time in rewarded crop fields compared with unrewarded fields [64]. Similarly, combining MeSA with *Calendula officinalis* as a reward plant increased the persistence of the predator *Propylea japonica* in apple orchards, leading to long-term suppression of the aphid *Aphis citricola* compared with control orchards [65]. However, the effectiveness of reward resources depends on the feeding ecology of the natural enemy. Nectar and pollen may support adult parasitoids and omnivorous predators, whereas strictly carnivorous predators may depend more strongly on alternative prey, shelter, oviposition sites, and suitable microhabitats.

Despite their promise, there is a concern about potential habituation of natural enemies to synthetic HIPVs which may function as unreliable cues when they are deployed in the absence of actual herbivores or prey/hosts [61,67]. However, providing alternative food resources such as pollen and nectar may alleviate the concern [66,68]. In spring barley, *Hordeum vulgare*, the combination of HIPVs with flowering strips and overwintering shelters significantly increased recruitment of the green lacewing *Chrysoperla carnea* in agricultural landscapes. This led to a promising decrease in aphids (*Sitobion avenae, Rhopalosiphum padi*) populations below the economic damage threshold in barley [69]. Despite successful attraction of natural enemies to alternative floral resources, they may leave these resources without moving into the crop, suggesting that floral rewards alone may not always retain natural enemies within the target crop [43,70]. Natural enemy attraction should not be considered evidence of successful biological control unless it results in increased predation or parasitism, reduced pest density or crop damage, and ultimately improved yield or economic return. For example, MeSA lures in cranberry fields not only increased several beneficial arthropods and predation on sentinel eggs but also attracted herbivores and reduced spider abundance [71]. Similarly, reduced trap catches or increased natural enemy abundance may reflect behavioral manipulation without reducing the pest population [42]. Field trials should therefore measure sequential outcomes, including natural enemy arrival and retention, predation or parasitism, pest abundance, crop damage, yield, pesticide use, and economic feasibility.

### 4.5. Improving Semiochemical-Based Push–Pull Strategies

The push–pull strategy is an established IPM approach that uses behavioral manipulation to reduce pest pressure, decrease reliance on synthetic pesticides, and improve the sustainability of agricultural systems [72]. This approach primarily manipulates pest behavior by using repellent cues to drive pests away from the main crop and attractive cues to concentrate them in trap crops or baited traps; in some systems, it may also enhance natural enemy activity [73]. The push–pull strategy works by repelling insect pests away from the main crop using repellent plants or semiochemicals, while attracting them toward more attractive trap crops or baited traps, where pest populations can be concentrated and managed (Figure 2B). For example, in southern and eastern Africa, push–pull systems have been used to manage maize stemborers, including *Eldana saccharina*, *Chilo partellus*, *Sesamia calamistis*, and *Busseola fusca.* Napier grass (*Pennisetum purpureum*) is planted along crop borders as a trap crop that provides the pull effect, while silverleaf desmodium (*Desmodium uncinatum*) is intercropped with maize (*Zea mays*) to repel stemborer moths from the main crop [74]. Recent studies have shown that volatile compounds released by companion plants, including β-caryophyllene, DMNT, MeSA, indole, (E)-2-hexenal, (Z)-3-hexenyl acetate, and (E)-β-farnesene, may contribute to pest repellence, plant defense signaling, and attraction of stemborer parasitoids in maize systems [75,76]. This suggests that the technique’s success is partially attributable to the recruitment of natural enemies [77,78,79]. Recent developments in push–pull strategies have focused on semiochemical-based approaches, such as using the anti-aggregation pheromone verbenone as a push component to repel ambrosia beetles in avocado, combined with ethanol-baited traps as the pull component [80]. This approach reduced beetle densities within the crop and increased captures in baited traps. The identification and maintenance of plants that emit diverse volatile compounds are crucial for inter/trap cropping and VOC mediated plant-to-plant interaction. However, large-scale field trials and careful selection of semiochemicals are needed to ensure that push–pull systems suppress target pests without disrupting natural enemy recruitment, repelling beneficial insects, or causing unintended non-target effects.

## 5. Environmental and Ecological Modifiers of Chemical Signaling

Under natural environmental conditions, plants experience multiple stresses. The release of VOCs, including HIPVs, represents an inducible plant defense that can mediate responses to herbivores, pathogens, and abiotic stress while also influencing neighboring plants and natural enemies [81]. The production and release of plant volatiles are altered by global climate change factors, such as rising atmospheric CO_2_ concentrations, elevated tropospheric ozone (O_3_), and increasing temperatures [82]. These factors can affect the biosynthesis, emission rate, atmospheric stability, and composition of herbivore-induced VOCs, thereby influencing herbivore performance and natural enemy foraging success throughout the plant life cycle [83]. Abiotic stressors can reduce the attractiveness of herbivore-infested plants to natural enemies. For example, water stress and elevated CO_2_ may alter stomatal conductance, plant physiology, and defense signaling, leading to reduced or modified HIPV emission [81,84]. Similarly, elevated atmospheric O_3_ can degrade reactive volatile compounds, including some terpenoids that are important cues for parasitoid attraction [85]. In addition, plants growing in nutrient-deficient soils may show reduced HIPV emission [86]. Moreover, under drought stress, parasitoid wasps may lose attraction to volatiles emitted by herbivore-infested plants [87]. Certain stress conditions can also affect natural enemy feeding efficiency, development, or reproduction by altering the quality of their prey or hosts [88].

Anthropogenic land use change and pollution may further modify plant–herbivore–natural enemy interactions. Urban warming has been shown to increase the abundance of some herbivorous pests; for example, warmer urban sites supported higher densities of scale insects on street trees [89]. Urbanization and habitat fragmentation may also alter the abundance, diversity, and spatial distribution of predators and parasitoids, potentially weakening biological control services in highly modified landscapes [90,91]. Air pollution may influence insect communities directly or indirectly through effects on host plants and atmospheric chemical cues. For example, herbivore abundance in urban green spaces was positively associated with atmospheric carbon monoxide (CO) and nitrogen dioxide (NO_2_) concentrations, although these observational relationships do not necessarily demonstrate causation [92,93]. Pollutants such as O_3_ may also degrade or transform volatile compounds used by herbivores and natural enemies, thereby disrupting chemically mediated interactions. Collectively, these findings suggest that the effectiveness of CBC may be compromised or altered by climatic and other anthropogenic environmental factors (Figure S1).

## 6. Complementary Ecological, Breeding, and Genetic Strategies

### 6.1. Habitat Management for Natural Enemy Conservation and Retention

Many agroecosystems provide unfavorable environments for natural enemies because of frequent disturbance, simplified vegetation structure, and limited availability of alternative resources [94]. These resources need to be incorporated into the agricultural landscape to meet the spatial and temporal needs of natural enemies while also being practical and feasible for farmers. Habitat management is an ecologically based CBC approach aimed at improving the availability of key resources for natural enemies, including food, shelter, alternative prey or hosts, and oviposition sites, thereby enhancing biological control within cropping systems [95]. In CBC habitat management studies, non-native plant species have often been used, whereas native plants may provide several advantages, including adaptation to local conditions, lower maintenance requirements, long-term habitat provision for natural enemies and pollinators, and support for biodiversity conservation [96,97,98]. A recent study demonstrated the potential of Australian native flowering plants, such as *Mentha satureioides* and *Lotus australis*, to support natural enemy abundance in brassica systems compared with an exotic plant and a naturally occurring weedy grass [99]. *M. satureioides* promoted effective parasitism and reduced pest densities. An important implementation to improve and conserve natural enemy density and diversity in agricultural landscapes is to combine shelter provision with native perennial flowering plant intercropping to improve natural enemy movement and promote faster response towards pest colonization [100,101]. Shelter habitats located close to pest-infested crop areas may enhance biological control, although optimal distances are likely to depend on crop type, pest species, natural enemy dispersal ability, and shelter design [102,103]. Field experiments must be conducted on various crops, pest species and shelter types at different distances from the infested crop to evaluate distance-dependent effects of shelters to accommodate natural enemies for effective biological control strategies. The value of plant-derived food resources depends on the feeding ecology of the natural enemy: nectar, pollen, extrafloral nectar, honeydew, and guttation may strongly benefit adult parasitoids and omnivorous predators, whereas strictly carnivorous predators may rely more on alternative prey, shelter, and suitable oviposition or nesting sites [104]. Understanding the quality and availability of these plant-derived food sources is essential for the success of biological pest control strategies [105,106]. However, the presence of these food sources can vary considerably, making their availability unpredictable for a foraging insect [107]. Another under-exploited resource is plant guttation, an exudation fluid secreted at the margins and tips of leaves through pores, which is a nutrient-rich food source for natural enemies (Figure 2C) [108]. Alongside habitat-based measures that support natural enemies, pest pheromone-based mating disruption can provide a complementary IPM strategy by interfering with mate location and reducing pest reproduction and subsequent crop colonization (Figure 2D). A study on plant guttation in highbush blueberry fields highlighted its nutritional value and explored that guttation was present throughout the day and entire growing season, providing a reliable food source to support biological control agents population [109]. However, guttation may also have negative effects if droplets contain pesticide residues or toxic secondary metabolites, vary substantially in nutritional quality, or provide resources that support pest survival and increase crop damage [110]. These negative factors of guttation should be carefully evaluated if it is used as a food source.

### 6.2. Balancing Plant Resistance Traits with Natural Enemy Performance

In response to herbivory, plants employ constitutive and inducible defenses, including physical barriers, toxic or deterrent secondary metabolites, defensive proteins, and volatile cues that can reduce herbivore damage [111]. However, these defenses are not always target specific; they may affect not only herbivore performance but also the behavior and effectiveness of natural enemies [112]. Physical defenses, such as dense or sticky trichomes, can impede predator mobility, increase foraging time, and reduce biological control effectiveness [113]. Furthermore, plant chemical defense can impair the performance of natural enemies, particularly when defensive compounds are transferred through herbivores that sequester plant metabolites for their own defense [114,115]. Some natural enemies can tolerate or exploit plant defensive compounds acquired through their prey, which may enhance their survival or defense against higher-order predators. For instance, the lady beetle *Coccinella septempunctata* can accumulate plant-derived pyrrolizidine alkaloids acquired through prey and use them in defense against its own predators [116]. A recent study on the green lacewing *Chrysoperla carnea* demonstrated tolerance or adaptation to the plant secondary metabolite isothiocyanate [117].

Integrating plant resistance traits to balance plant defenses against herbivores while supporting the natural enemies’ retention is a strategic approach towards sustainable pest management and environmental stability. Rather than maximizing plant resistance alone, future strategies should aim to combine herbivore-resistant crop traits with natural enemy compatibility. This may involve selecting or conserving natural enemies that tolerate plant defensive compounds, while avoiding plant traits that strongly impair predator or parasitoid mobility, survival, or reproduction [118]. This resilience ensures their retention in the cropping system while reducing the reliance on chemical pesticides and promoting a balanced sustainable agroecosystem.

### 6.3. Integrating Selective Breeding and Genetic Engineering to Enhance CBC

Genetic modification can provide tools to incorporate direct and indirect defense traits into crop plants, potentially reducing reliance on repeated pesticide applications [119]. Genetic engineering has been used to manipulate VOC production in crop plants, with the aim of repelling herbivores or enhancing the attraction of natural enemies [120,121,122] (Figure 2E). Experimental studies with transgenic tobacco and rice have shown that engineered production of defensive terpenoids can reduce damage by the rice pest *Chilo suppressalis* by attracting parasitoid wasps [123]. Similarly, *Arabidopsis thaliana* plants overexpressing linalool and its derivatives significantly repelled aphid attraction [124]. Genetic manipulation of maize with an *(E)-β-caryophyllene* synthase gene reduced root damage and adult emergence of western corn rootworm compared with non-modified plants [125]. More recently, CRISPR/Cas9 knockout of the cotton genes *GhCYP82L1* and *GhCYP82L2* eliminated DMNT emission, whereas overexpression of DMNT biosynthesis genes increased herbivore-induced volatile emission and recruitment of the parasitoid *Peristenus spretus*, demonstrating the potential for precise manipulation of volatile-mediated crop defense [126]. A key limitation is that constitutive production of defensive volatiles or toxic metabolites may impose metabolic costs on plant growth, development, reproduction, or yield and may also alter interactions with pollinators, herbivores, and natural enemies. Therefore, inducible, tissue-specific, or herbivore-responsive expression systems may be preferable to continuous expression. However, despite these promising results, an important consideration is to ensure that modified plants release these chemical cues in response to herbivory attack. For instance, if a cue is persistently emitted in the absence of herbivores, natural enemies may learn to ignore it as an unreliable signal, reducing its effectiveness and potentially altering non-target interactions. Therefore, extensive research is needed to determine the long-term effectiveness of this technique [43,127].

One potential strategy is to use genetically engineered plants as companion plants that attract and retain natural enemies within cropping systems. Genetically engineered plants for enhanced volatile production can be used as companion plants in push–pull strategy. Genetically modified plants could be used as trap crops for herbivores or as attractant plants for natural enemies, thereby reducing damage to the non-genetically engineered main crop [119]. Although genetic engineering may provide useful tools for modifying plant volatile emissions, its application requires careful evaluation of long-term ecological effects, gene flow to conventional or wild relatives, non-target impacts, regulatory constraints, and public acceptance. Where these concerns limit adoption, selective breeding and non-transgenic semiochemical or habitat management approaches should be prioritized as alternative strategies.

## 7. Summary and Future Perspectives

Within the defined context of crop agroecosystems, previous research on CBC and chemical ecology has focused particularly on HIPVs, semiochemical-mediated natural enemy recruitment, and push–pull strategies. In this review, we expand this perspective to include the integration of HIPVs with natural enemy pheromones and other insect-derived cues, habitat management combined with the provision and conservation of plant-derived food resources, plant defense traits in breeding, and infochemical-based genetically modified crops (Figure 2E). Despite many promising examples, important knowledge gaps remain regarding the specificity, reliability, and ecological consequences of chemical signals used in CBC, particularly under climate change scenarios. Future research should therefore focus on identifying the right chemical signals in the right ecological contexts, ensuring that attraction translates into natural enemy retention, increased predation or parasitism, pest suppression, and improved crop protection. If chemical cues attract non-target organisms, repel key natural enemies, or fail to match prey/host availability, they may reduce the effectiveness of biological control strategies. Moreover, attraction of natural enemies does not necessarily lead to increased predation or parasitism, reduced pest populations, or improved crop yield. Practical adoption may also be constrained by lure costs, formulation stability, repeated deployment, labor requirements, and inconsistent performance across crops and environments. Future studies should therefore evaluate ecological effectiveness together with non-target effects, economic feasibility, pesticide reduction, and yield benefits.

## Data Availability

No new data were created or analyzed in this study. Data sharing is not applicable to this article.

## Supplementary Materials

The following supporting information can be downloaded at: https://www.mdpi.com/article/10.3390/insects17080808/s1. Table S1: Examples of synthetic plant volatiles and insect pheromones evaluated for recruiting or retaining natural enemies in crop and managed-plant systems. Figure S1: Functions of plant volatile organic compounds and factors influencing their emission. Biotic and abiotic factors can have complex direct and indirect effects on HIPV formation, emission, stability, and ecological function. Plant VOCs contribute to several ecological processes, including direct and indirect defense against herbivores and pathogens, attraction of pollinators and seed dispersers, and VOC-mediated plant–plant signaling in response to herbivory. Circle 1 represents abiotic factors, whereas circle 2 represents biotic factors. The studies supporting the examples summarized in Table S1 are cited in the table and are included in the main reference list [71,128,129,130,131,132,133,134,135,136,137,138,139,140,141,142,143,144,145,146,147,148,149,150,151,152,153,154].
