# Supplementary material for: Chemical Signaling and Integrated Strategies for Biological Control in Crop Agroecosystems
Source: Insects. 2026 Aug 4;17(8):808. doi: 10.3390/insects17080808 (PMC13513163; doi:10.3390/insects17080808)
Supplement: Supplementary file 1 [file insects-17-00808-s001.zip › insects-4425473-supplementary.pdf]

Table S1. Examples of synthetic plant volatiles and insect pheromones evaluated for recruiting or retaining natural enemies in crop and managed-plant systems.

Semiochemical or lure	Associated pest/host	Responding natural enemy	Plant system and experimental context	Reference
A. Plant-derived volatiles and plant-defense elicitors				
DMNT [(E)-4,8-dimethyl-1,3,7-nonatriene]	Elm leaf beetle, *Xanthogaleruca luteola* (egg-infested elm)	Egg parasitoid *Oomyzus gallerucae* (Eulophidae)	Field elm, *Ulmus minor*; field and behavioral assays	[128]
m-Cymene + 4′-ethylacetophenone	Mirid bug, *Apolygus lucorum*	Parasitoid *Peristenus spretus* (Braconidae)	Cotton field; lure deployment and parasitism assessment	[129]
(E)-β-Ocimene and (E,E)-α-farnesene	Asparagus pest complex, including *Crioceris asparagi* and *Ophiomyia simplex*	Parasitoid wasps, including Pteromalidae and Braconidae	Asparagus field; lures attracted parasitoids, with limited evidence for increased parasitism	[130]
Indole	No single focal prey specified	Lady beetles *Propylaea japonica* and *Harmonia axyridis*; *Chrysopa* spp.	Cotton fields; two-year trapping study	[131]
Linalool	No single focal prey specified	Lady beetle *Propylaea japonica*	Cotton fields; two-year trapping study	[131]
α-Pinene and β-pinene	No single focal prey specified	Green lacewing *Chrysoperla sinica*; *Orius* spp. and *Chrysopa* spp. in compound- and year-dependent responses	Cotton fields; two-year trapping study	[131]
Methyl jasmonate	No single focal prey specified	Predatory bugs *Orius* spp.	Cotton fields; two-year trapping study	[131]
Dimethyl disulfide	Cabbage root fly, *Delia radicum*	Rove beetles *Aleochara bilineata* and *A. bipustulata*; other ground predators	Cabbage, *Brassica oleracea*; field evaluation	[132]
Benzaldehyde	No single focal prey specified	Green lacewing *Chrysoperla plorabunda*	Washington apple orchards; field trapping	[133]
Iridodial	No single focal prey specified	Green lacewings *Chrysopa nigricornis* and *C. oculata*	Washington apple orchards; field trapping	[133]
Methyl salicylate + iridodial	No single focal prey specified	Green lacewings *Chrysopa nigricornis* and *C. oculata*	Washington apple orchards; field trapping	[133]
Squalene	No single focal prey specified	Male *Chrysopa nigricornis*	Washington apple orchards; field trapping	[133]
cis-α-Bergamotene	*Manduca sexta* eggs/larvae on wild tobacco	*Geocoris* spp. predators	Wild tobacco, *Nicotiana attenuata*; field predation and behavioral assays	[134]
Pregeijerene [1,5-dimethylcyclodeca-1,5,7-triene]	Citrus root weevil, *Diaprepes abbreviatus*	Naturally occurring entomopathogenic nematodes	Citrus; belowground field assays	[135]
Aphid-induced volatile blend, including 3-ethylacetophenone, 1,2-diethylbenzene, α-pinene and butyl acrylate	Aphids on several host plants, including *Aphis gossypii*	Lady beetle *Harmonia axyridis*	Laboratory electrophysiology and olfactometer assays	[136]
45% isopropanol	No focal prey specified	Adult female green lacewing *Chrysopa quadripunctata*	Unsprayed apple orchard; field trapping	[137]
Methyl salicylate	Soybean aphid, *Aphis glycines*	Lady beetle *Coccinella septempunctata*	Soybean; laboratory behavioral assays	[138]
2-Phenylethanol	No single focal prey specified	Syrphid flies, including *Eupeodes* spp.	Apple, pear and walnut orchards; field monitoring study	[139]
(Z)-3-Hexenyl acetate and α-farnesene	Tarnished plant bug, *Lygus lineolaris* (eggs)	Egg parasitoid *Anaphes iole* (Mymaridae)	Cotton; behavioral and host-attack assays	[140]
Nonanal	No single focal prey specified	Lady beetle *Harmonia axyridis*	Flowers of *Sophora japonica*; field trapping and laboratory assays	[141]
Methyl salicylate	No single focal prey specified	*Geocoris pallens*, syrphid flies and *Stethorus punctum picipes*	Washington hop yard; field trapping	[142]
(Z)-3-Hexenyl acetate	No single focal prey specified	*Deraeocoris brevis*, *Orius tristicolor* and *Stethorus punctum picipes*	Washington hop yard; field trapping	[142]
α-Pinene	Green peach aphid, *Myzus persicae*	Parasitoid *Aphelinus varipes*	Chili pepper, *Capsicum annuum*; laboratory olfactometer assay	[143]
(E)-β-Ocimene	Green peach aphid, *Myzus persicae* (peach-associated volatile context)	Larvae of *Adalia bipunctata* and *Chrysoperla carnea*	Laboratory navigation assays using peach-associated synthetic volatiles	[144]
β-Myrcene + β-caryophyllene	Whitefly, *Bemisia tabaci*	Parasitoid *Encarsia formosa*	Tomato glasshouse; dispenser and biological-control assessment	[145]
Methyl salicylate	Cranberry arthropod community; no single focal prey	Beneficial arthropod community, including predators and syrphid flies	Cranberry fields; field trapping and sentinel-prey assessment	[71]

Semiochemical or lure	Associated pest/host	Responding natural enemy	Plant system and experimental context	Reference
B. Insect pheromones used directly or exploited as kairomones				
Methyl (2E,4Z)-decadienoate (male-produced aggregation-pheromone component)	Consperse stink bug, *Euschistus conspersus*	Tachinid parasitoid *Gymnoclytia occidentalis*	Apple orchards; field trapping	[146]
(R)-(+)-γ-Decalactone (sex pheromone)	Hermit beetle, *Osmoderma eremita*	Predatory click beetle *Elater ferrugineus*	Oak, *Quercus robur*; forest/conservation system	[147]
(2E,5R,6E,8E)-5,7-Dimethyl-2,6,8-decatrien-4-one (female sex pheromone)	Pine bast scale, *Matsucoccus josephi*	*Elatophilus hebraicus* and brown lacewing *Hemerobius stigmaterus*	Pine forest; pheromone-baited traps	[148]
(2E,4E,6R,10R)-4,6,10,12-Tetramethyl-2,4-tridecadien-7-one (female sex pheromone)	Pine bast scale, *Matsucoccus matsumurae*	Brown lacewing *Hemerobius stigma* and *Elatophilus* spp.	Pine forest; pheromone-baited traps	[149]
(3S,7R,8E,10E)-3,7,9-Trimethyl-8,10-dodecadien-6-one (female sex pheromone)	Pine bast scale, *Matsucoccus feytaudi*	*Elatophilus crassicornis*, *E. nigricornis* and *Hemerobius stigma*	Maritime pine, *Pinus pinaster*; forest trapping studies	[148,149]
(Z)-16-Methyl-9-heptadecenyl isobutyrate (female sex-pheromone component)	Tussock moth, *Euproctis taiwana*	Phoretic egg parasitoid *Telenomus euproctidis*	Field trapping; host plant not specified in the cited study	[150]
Mountain pine beetle pheromone lure, including trans-verbenol and exo-brevicomin	Mountain pine beetle, *Dendroctonus ponderosae*	Predators and competitors, including *Thanasimus dubius*	Wisconsin red-pine forests, *Pinus resinosa*; anticipatory-biological-control trapping	[151]
Male-produced aggregation-pheromone blend: (E)-2-hexenal, α-terpineol, linalool, terpinen-4-ol and benzyl alcohol	Colorado potato beetle, *Leptinotarsa decemlineata* (management target)	Predatory stink bug *Podisus maculiventris*	Potato; pheromone-mediated predator augmentation	[152]
(E)-2-Octenal + (E)-2,7-octadienal (female-produced sex-pheromone blend)	*Orius sauteri*	Male *Orius sauteri*	Field bioassay; crop was not specified in the cited paper	[153]
(E)-2-Hexenal + (E)-2-hexen-1-ol + benzaldehyde + benzyl alcohol (male-produced aggregation-sex pheromone)	*Arma chinensis*	Adult *Arma chinensis* of both sexes	Poplar/managed-forest system; field verification	[154]

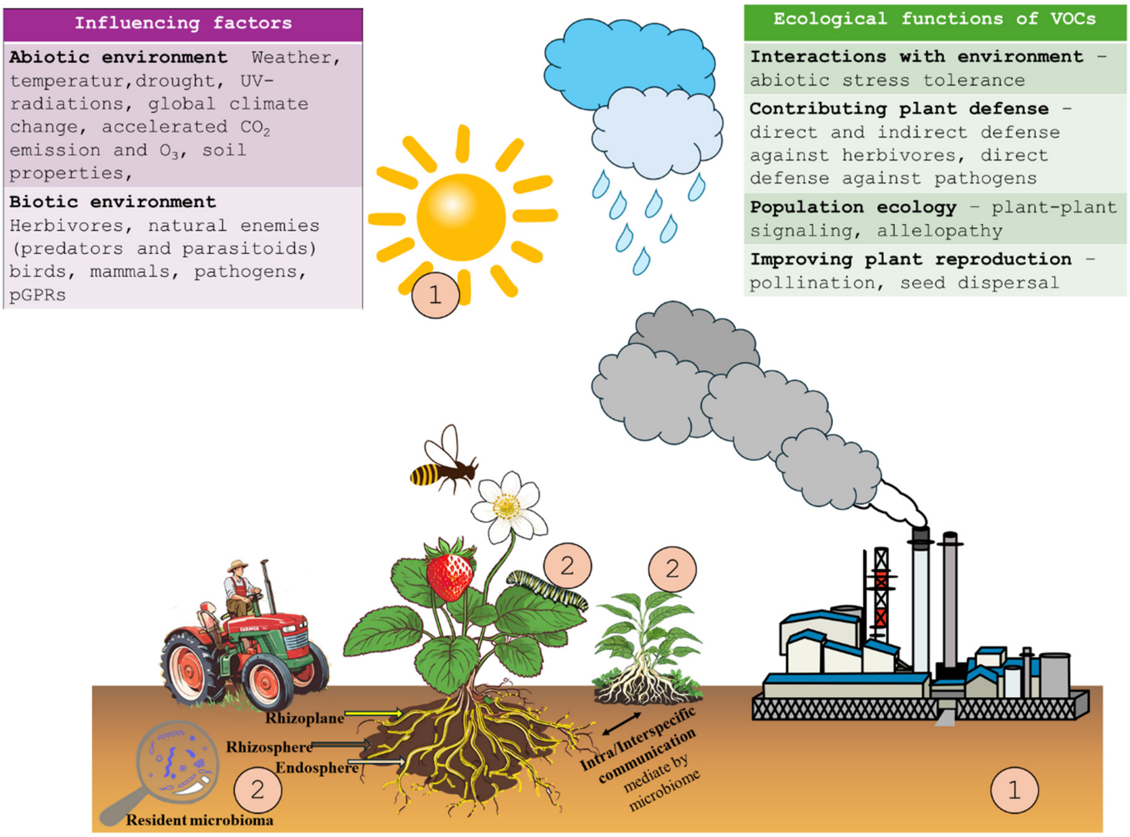

Figure S1. Functions of plant volatile organic compounds and factors influencing their emission. Biotic and abiotic factors can have complex direct and indirect effects on HIPV formation, emission, stability, and ecological function. Plant VOCs contribute to several ecological processes, including direct and indirect defense against herbivores and pathogens, attraction of pollinators and seed dispersers, and VOC-mediated plant–plant signaling in response to herbivory. Circle 1 represents abiotic factors, whereas circle 2 represents biotic factors.
